# Skeletal muscle-derived musclin attenuates glycolysis, oxidative stress, and pulmonary hypertension through the NPR3/AKT/mTORC1 pathway: Musclin in pulmonary hypertension

**DOI:** 10.3724/abbs.2024214

**Published:** 2024-12-04

**Authors:** Xiongshan Sun, Jia Wang, Yi Xiao, De Li, Qiang Wang, Wei Guo, Yongjian Yang

**Affiliations:** 1 Department of Cardiovascular Medicine the General Hospital of Western Theater Command Chengdu 610083 China; 2 Department of Laboratory Medicine the General Hospital of Western Theater Command Chengdu 610083 China; 3 Department of Pharmacy Stomatological Hospital of Chongqing Medical University Chongqing 400015 China

**Keywords:** musclin, PASMC, PH, mTORC1, NPR3, glycolysis, oxidative stress

## Abstract

Exercise ameliorates pulmonary hypertension (PH) progression. However, the underlying mechanisms are largely unclear. Musclin is an exercise-responsive myokine that exerts protective effects on cardiovascular diseases. The current study aims to explore the role of musclin in the development of PH. A monocrotaline (MCT)-induced mouse PH model is established. Adeno-associated virus serotype 6 (AAV6)-mediated gene transfer is used to induce musclin overexpression in skeletal muscle. Ultrasound and morphological analyses are utilized to assess the severity of PH. Cell viability assay, Ki-67 immunofluorescence staining, wound healing assay, and transwell assay are used to evaluate the proliferation and migration of pulmonary arterial smooth muscle cells (PASMCs). We find that the musclin levels in both plasma and skeletal muscle are decreased in MCT-treated mice. The external expression of musclin in skeletal muscle ameliorates pulmonary arterial remodeling and right ventricular dysfunction. *In vitro*, musclin treatment suppresses hypoxia-induced glycolysis, oxidative stress, proliferation, and migration. Further experiments reveal that musclin inhibits mechanistic target of rapamycin complex 1 (mTORC1) activity in hypoxia-stimulated PASMCs and pulmonary arteries of MCT-treated mice. Reactivating mTORC1 abolishes the protective role of musclin against PH. Additionally, musclin enhances its interaction with natriuretic peptide receptor 3 (NPR3) in PASMCs. Silencing of *NPR3* reverses the inhibitory effects of musclin on AKT phosphorylation, mTORC1 activity, glycolysis, oxidative stress, proliferation, and migration in hypoxia-challenged PASMCs. In conclusion, our study highlights the inhibitory role of musclin in the proliferation and migration of PASMCs and PH progression, thereby providing a novel potent therapeutic strategy for treating PH and partly clarifying the mechanism of exercise-mediated protection against PH.

## Introduction

Vascular remodeling of the pulmonary artery (PA) is considered a critical part of PH progression [1]. Current treatments focus mainly on reducing the resistance of PAs by inducing pulmonary vascular dilation. However, the therapeutic effects are not satisfactory, as PA remodeling is not ameliorated [2]. After endothelial dysfunction caused by adverse stress, such as hypoxia, growth and inflammatory factors, pulmonary arterial smooth muscle cells (PASMCs) migrate from the media to the intima, rapidly proliferate, and ultimately result in PA remodeling [3]. As the major component of the vascular wall, the reprogramming of PASMCs, which is characterized by excessive proliferation and migration, plays a critical role in the development of PH. Therefore, focusing on the mechanisms underlying PASMC reprogramming is highly important.

Accumulating evidence has demonstrated that exercise may reduce the mean pulmonary arterial pressure and morbidity of adverse events in PH patients [ 4, 5] . The beneficial effects of exercise on PH partly rely on skeletal muscle-secreted myokines [ 6, 7] . Musclin, also known as osteocrin, is an exercise-responsive myokine that is highly homologous to natriuretic peptides [8]. The protective roles of musclin in the cardiovascular system have been well investigated. Musclin is essential for exercise-induced cardiac protection and can prevent heart failure [ 9, 10] . The circulating musclin level is decreased in patients with hypertension and transcatheter aortic valve implantation [ 11, 12] , suggesting that musclin may play a protective role in vascular events. Additionally, Musclin has been confirmed to suppress the attachment of vascular endothelial cells to monocytes, which serves as a critical event during atherosclerosis [13]. However, little is known about the regulatory effect of musclin on pulmonary hypertension.

Although musclin can inhibit the proliferation of fibro-adipogenic progenitors [14], whether musclin affects the proliferation and migration of PASMCs is largely unknown. A previous study demonstrated that exercise benefits pulmonary and cardiac function and structure in PH partly by reducing oxidative stress [15]. Excessive oxidative stress plays a vital role in the proliferation and migration of PASMCs and the development of PH [16]. However, whether oxidative stress is involved in musclin-mediated regulation of PASMCs remains unclear. Increased glycolysis in vascular diseases plays an important role in promoting oxidative stress [17]. Metabolic reprogramming favouring glycolysis is an essential process for hypoxia-induced proliferation and migration of PASMCs and PH [18]. There are several lines of evidence that musclin suppresses glucose uptake and metabolism in adipose tissue and skeletal muscle [ 19, 20] . However, whether musclin affects local vascular glycolysis within the PA during PH has rarely been investigated.

In the present study, the levels of musclin in both skeletal muscle and the circulation in a monocrotaline (MCT)-induced mouse model of PH were decreased. Elevating muscline levels via adeno-associated virus serotype 6 (AAV6)-mediated skeletal muscle gene transfer (AAV- *Musclin*) significantly prevented PH development. Furthermore, musclin treatment *in vitro* suppressed hypoxia-induced proliferation, migration, oxidative stress, and glycolysis. Mechanistically, we demonstrated that musclin inhibits AKT/mechanistic target of rapamycin complex 1 (mTORC1) signaling by binding to natriuretic peptide receptor 3 (NPR3). Our findings suggested that skeletal muscle-derived musclin may serve as a potent therapeutic method for preventing PH.

## Materials and Methods

### Animals

C57BL/6J mice were purchased from Dashuo Animal Science and Technology (Chengdu, China). Smooth muscle cell-specific Tuberous sclerosis complex ( *Tsc1*)-knockdown ( *Tsc1* ^KD^; *Tsc1* ^fl/fl^sm22cre ^+/−^) mice were obtained from Jackson Laboratories (Cambridge, USA). All experimental procedures were approved by the Institutional Animal Care and Use Committee and the Ethics Committee of the General Hospital of Western Theater Command (2022EC2-ky037). The mice were kept at room temperature with a 12-h light/dark cycle and free access to water and food. To establish the mouse PH model, male C57BL/6J mice (8–12 weeks old) were subjected to MCT (60 mg/10 g body weight; Sigma, St Louis, USA) for 8 weeks with one administration every week as previously described [21]. Another mouse PH model that mimics clinical PH caused by hypoxia was established as described in a previous study [22]. Briefly, 8-week-old mice were subcutaneously injected with SU5416 (20 mg/kg; Sigma) once a week and maintained under chronic hypoxia (10% O _2_) for another 3 weeks. After the acquisition of hemodynamic parameters, the mice were anesthetized with pentobarbital (100 mg/kg) and then decapitated for sacrifice.

### Hemodynamic and ultrasound analysis

The mice were temporarily anaesthetized with pentobarbital (30 mg/kg) via intraperitoneal injection. The right ventricular systolic pressure (RVSP) was determined by using a pressure transducer catheter (Millar Instruments, Houston, USA). The right ventricular hypertrophy index (RVHI) was evaluated through the right ventricle weight-to-(left ventricle+interventricular septum) weight ratio. Pulmonary artery acceleration time (PAT), pulmonary artery ejection time (PET), right ventricular internal diameter (RVID), and right ventricular free wall thickness (RVFWT) were evaluated via the Vevo 2100 high-resolution imaging system (FUJIFILM VisualSonics, Toronto, Canada).

### Immunohistochemistry analysis

Morphological analysis of PAs was performed via standard hematoxylin and eosin (H&E) and α-SMA immunohistochemical staining and Masson’s trichrome staining as described previously [23]. The lung tissues were fixed, embedded and cut into 4-μm sections. The sections were then stained with hematoxylin and eosin or Masson staining reagents. For α-SMA immunohistochemical staining, lung sections were first washed with phosphate-buffered saline (PBS) and blocked with 5% fetal bovine serum. The sections were subsequently incubated with a primary antibody against α-SMA (1:800; Proteintech, Wuhan, China) at 4°C overnight and then incubated with the corresponding secondary antibody (1:10,000; Solarbio, Beijing, China) before being counterstained with Mayer’s hematoxylin for another hour. Image-Pro Plus software (NIH, Bethesda, USA) was used to analyse the images.

### Enzyme-linked immunosorbent assay (ELISA) of the serum musclin level

The serum musclin level was measured by using a mouse Osteocrin ELISA kit (Biomatik, Ontario, Canada) according to the manufacturer’s instructions. In brief, the serum samples were centrifugated at 1000 *g* for 15 min after being clotted for 2 h at room temperature. A total of 100 μL of samples and standard were added to each well. The wells were then covered with the adhesive strip and incubated for 2 h at 37°C. The liquid of each well was removed. The wells were added with 100 μL of biotin-antibody (1×), covered with the adhesive strip and incubated for 1 h at 37°C. Subsequently, the wells were cleaned, washed, added with 100 μL of HRP-avidin (1×), and then incubated for 1 h at 37°C. After washing, the wells were then added with 90 μL of TMB substrate and incubated in dark for 30 min at 37°C. Finally, 50 μL of stop solution was added to each well. The OD value at 450 nm for each well was determined within 5 min through a microplate reader.

### AAV-6 transfection *in vivo*

The *Musclin* cDNA was Myc-tagged and integrated into the pdsMCKE vector downstream of the promoter of muscle creatine kinase to produce AAV- *Musclin*. AAV- *Musclin* (5 × 10 ^11^ pfu/mL; 50 μL) was injected into the right quadriceps muscle of C57BL/6J mice via intramuscular administration.

### Cell culture and treatment

Primary PASMCs were obtained from PAs of C57BL/6J mice (8–12 weeks old) as described in a previous study [24]. Briefly, the adventitia and endothelia of PAs were removed, and the media were cut into 1-mm ^3^ pieces to obtain PASMCs. PASMCs were cultured in a humidified 5% CO _2_ incubator at 37°C under normoxic (74% N _2_ and 21% O _2_) or hypoxic (92% N _2_, and 3% O _2_) conditions. Musclin (Abcam, Cambridge, UK) was used at doses of 0, 10, 20, 30, 40, 50, and 100 nM for 24 h [ 10, 13] . For siRNA transfection, the cells were incubated with *Tsc1* siRNA (si- *Tsc1*), *Tfr1* siRNA (si- *Tfr1*), or *Npr3* siRNA (si- *Npr3*) and Lipofectamine RNAiMAX Transfection Reagent (Invitrogen, Carlsbad, USA) for 8 h. The sequence of si- *Tsc1* was 5′-GCUUUGACUCUCCCUUCUA-3′ [25]. The sequence of si- *Npr3* was 5′-GCUCUACAGCGACGACAAA-3′ [10]. The sequence of si-transferrin receptor 1 ( *Tfr1*) was as follows: 5′-ACAAGUUAGAGAAUGCUGAUCUGGC-3′ [26]. The sequence of scramble siRNA was 5′-UGGUUUACAUGUCGACUAA-3′.

### Western blot analysis

Total protein was extracted from isolated quadriceps muscle, PAs of mice and PASMCs by using RIPA buffer (Beyotime, Shanghai, China). Western blot analysis was performed as described in our previous study [27]. Briefly, protein samples were separated via sodium dodecyl sulfate-polyacrylamide gels and then transferred to polyvinylidene fluoride membranes (Millipore, Billerica, USA). The membranes were subsequently blocked with 5% bovine serum albumin (BSA), incubated with the corresponding primary antibodies overnight at 4°C, and finally incubated with the corresponding secondary antibodies. The primary antibody against musclin was purchased from BioVendor (Brno, Czech Republic). The primary antibodies against glyceraldehyde-3-phosphate dehydrogenase (GAPDH), β-actin, PCNA, superoxide dismutase 1 (SOD1), TSC1, phosphor (p)-S6, S6, p-eukaryotic translation initiation factor 4E-binding protein 1 (4EBP1), 4EBP1, p-AKT, and AKT were purchased from Cell Signaling Technology (Danvers, USA). The primary antibody against NPR3 was purchased from Abcam. The primary antibodies against NADPH oxidase 4 (NOX4) and TFR1 were purchased from Proteintech. For the coimmunoprecipitation (co-IP) assay, the IP and input protein lysates were subjected to western blot analysis via primary antibodies against NPR3 and TFR1 (Proteintech). The development step was performed with a brand chemiluminescence imager (Azure 300; Azure Biosystems, Dublin, USA). The quantitative analysis was performed with ImageJ software.

### Cell Counting Kit-8 (CCK-8) assay

The viability of PASMCs was analyzed by using a CCK-8 kit (Beyotime) according to the manufacturer’s instructions. Briefly, PASMCs were cultured in a 96-well plate (0.5 × 10 ^4^ cells per well) and then incubated at 37°C with 10 μL of CCK-8 agent for 2 h. Finally, viability was measured by calculating the relative absorbance at 450 nm through a microplate reader.

### Immunofluorescence staining

After fixation and membrane disruption, the PASMCs were blocked with 5% BSA. Then, the PASMCs were incubated overnight with a primary antibody against Ki-67 (1:1000; Cell Signaling Technology) in the dark at 4°C and subsequently incubated with Alexa Fluor 488-conjugated goat anti-rabbit antibodies (1:3000; Molecular Probes Inc., Eugene, USA) in the dark for 1 h. Finally, the PASMCs were incubated with 4′,6-diamidino-2-phenylindole (DAPI; 5 mg/mL; VECTOR Labs, Burlingame, USA) for 5 s at room temperature. For dihydroethidium (DHE) staining, PASMCs were incubated at 37°C with DHE (5 mg/mL; Beyotime) for 45 min. Images were obtained and analyzed with a fluorescence microscope (Olympus, Tokyo, Japan).

### Wound healing assay

Prepared PASMCs were cultured in a 6-well plate (1 × 10 ^5^ cells per well). After serum deprivation for 12 h, a scratch was made in the middle area of the PASMCs via a 200-μL sterile pipette tip. Finally, the wound healing rate was determined via microscopic visualization.

### Transwell assay

PASMCs were cultured in transwell chambers with 8-μm pores (Millipore). The chambers were then placed onto 24-well plates filled with serum-free medium and cultured for 8 h. Subsequently, the PASMCs inside the chambers were removed, and the transmembrane PASMCs were fixed, washed, and stained with 1% crystal violet for 20 min. The number of migrated cells was determined from 5 randomly selected fields per chamber.

### Malonyldialdehyde (MDA) and glutathione (GSH) detection

The levels of MDA or GSH were determined by using a malonyldialdehyde assay kit (Cell Biolabs Inc., San Diego, USA) or a glutathione assay kit (Beyotime) according to the manufacturer’s instructions.

### Extracellular acidification rate (ECAR) analysis

The ECAR was determined as described in our previous study [27]. PASMCs were transferred to a Seahorse 96-well plate. Glucose, oligomycin, and 2-deoxy-D-glucose (2-DG) were added according to the manufacturer’s instructions. Glycolysis was calculated as (the last rate measurement before the application of oligomycin)-(minimum rate measurement before the addition of glucose). The glycolytic capacity was calculated as (maximum rate measurement after adding oligomycin)–(minimum rate measurement after application of 2-DG).

### Statistical analysis

Data were presented as the mean±SD. Unpaired Student’s *t* tests were performed for comparisons of 2 independent groups. Two-way analysis of variance (ANOVA) was performed for comparisons that involved two factors with corresponding *post hoc* tests. All tests in this study were two-tailed. *P*  < 0.05 was considered statistically significant.

## Results

### AAV6-mediated overexpression of musclin in skeletal muscle attenuates MCT-induced PH development in mice

**Figure 1:**
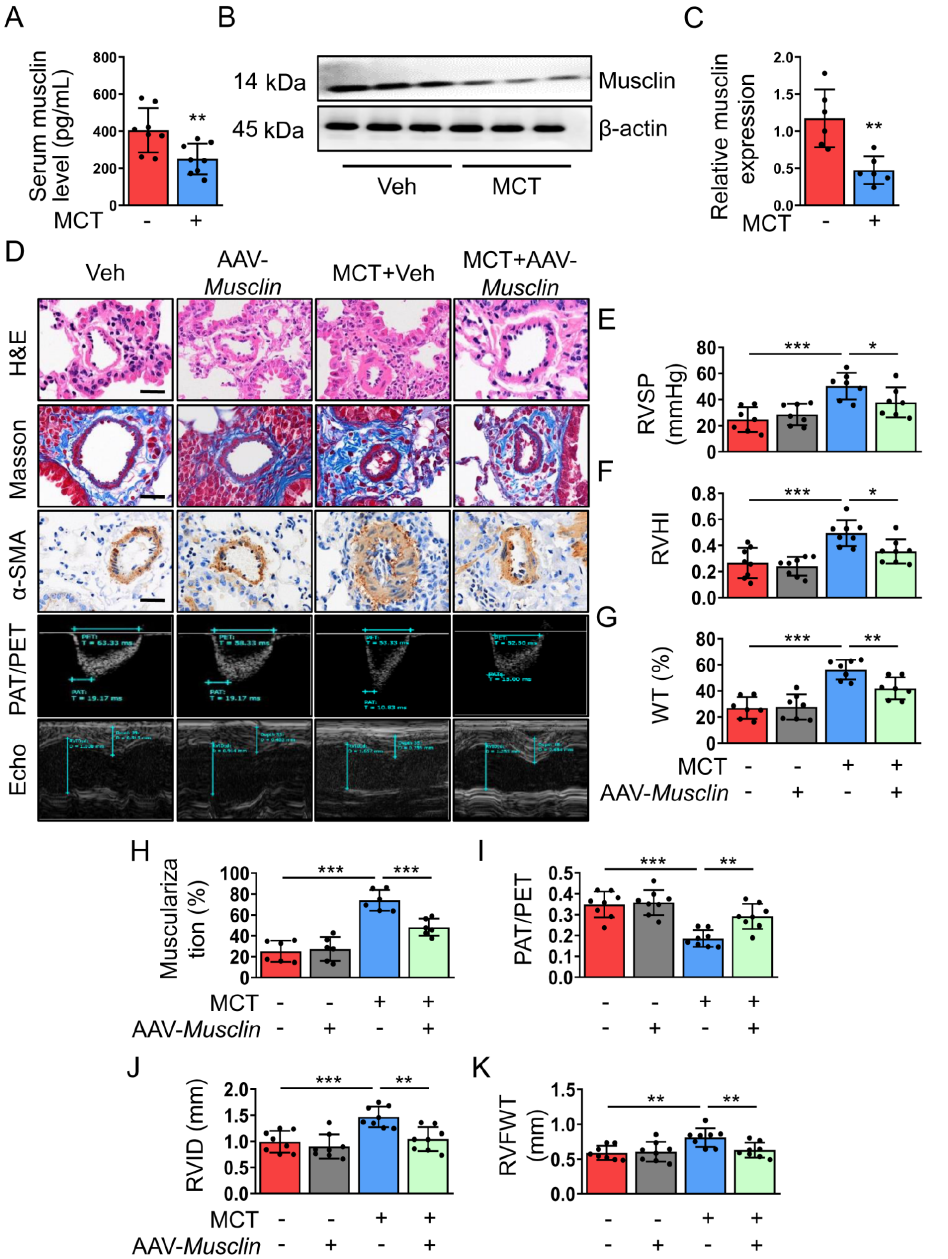
Figure 1 Skeletal muscle-derived musclin ameliorates pulmonary arterial remodeling and right ventricular hypertrophy in an experimental PH model (A) Serum musclin levels in mice in different groups were determined by ELISA (n = 8). (B,C) Representative western blots and corresponding quantification of musclin expression in right quadricep muscles from mice in different groups (n = 6). (D) Representative H&E staining images (scale bar: 20 μm), Masson staining images (scale bar: 20 μm), immunohistochemical staining images of α-SMA (scale bar: 20 μm), and echocardiography images of the mice in the different groups. (E–K) Quantification of (E) RVSP (n = 7), (F) RVHI (n = 8), (G) percentage of pulmonary wall thickness/total thickness (n = 7), (H) percentage of muscularization in PAs (n = 6), (I) PAT/PET (n = 8), (J) RVID (n = 8), and (K) RVFWT (n = 8) in the mice of different groups. *P < 0.05, **P < 0.01, and ***P < 0.001. Student’s t test for (A and C). Post hoc test for LSD in (E, F, G, H, I, J, and K).

To investigate whether skeletal muscle plays a paracrine/autocrine role during PH development, we first assessed the musclin levels in the quadriceps muscle and peripheral blood in an MCT-induced mouse model of PH. After 8 weeks of MCT treatment, we observed reduced musclin levels in the quadriceps muscle as well as in mouse serum ( Figure 1A–C). Since endogenous musclin was downregulated in the skeletal muscle and plasma of MCT-induced PH mice, we next determined whether musclin overexpression in skeletal muscle affects the progression of PH. AAV- *Musclin* carrying the muscle creatine kinase promoter was administered intramuscularly immediately after the first MCT injection to induce muscle-specific overexpression of musclin. Eight weeks after AAV- *Musclin* transfection, ELISA and western blot analysis revealed that the levels of musclin in the plasma and quadriceps muscle were both elevated ( Supplementary Figure S1A–C). Morphological, hemodynamic, and ultrasonic analyses revealed that eight weeks of MCT treatment led to increased RVSP, RVHI, PA wall thickness, vascular muscularization, RVID, and RVFWT. Moreover, PAT/PET was reduced after MCT stimulation. However, the abovementioned alterations in parameters induced by MCT were abolished after the overexpression of musclin in skeletal muscle ( Figure 1D–K). The protective effects of musclin were also verified in another SU5416/hypoxia-induced mouse PH model ( Supplementary Figure S2A–C). Taken together, these data suggest that musclin alleviates PH pathogenesis.

### Musclin mitigates the proliferation and migration of PASMCs after hypoxic stimulus

**Figure 2:**
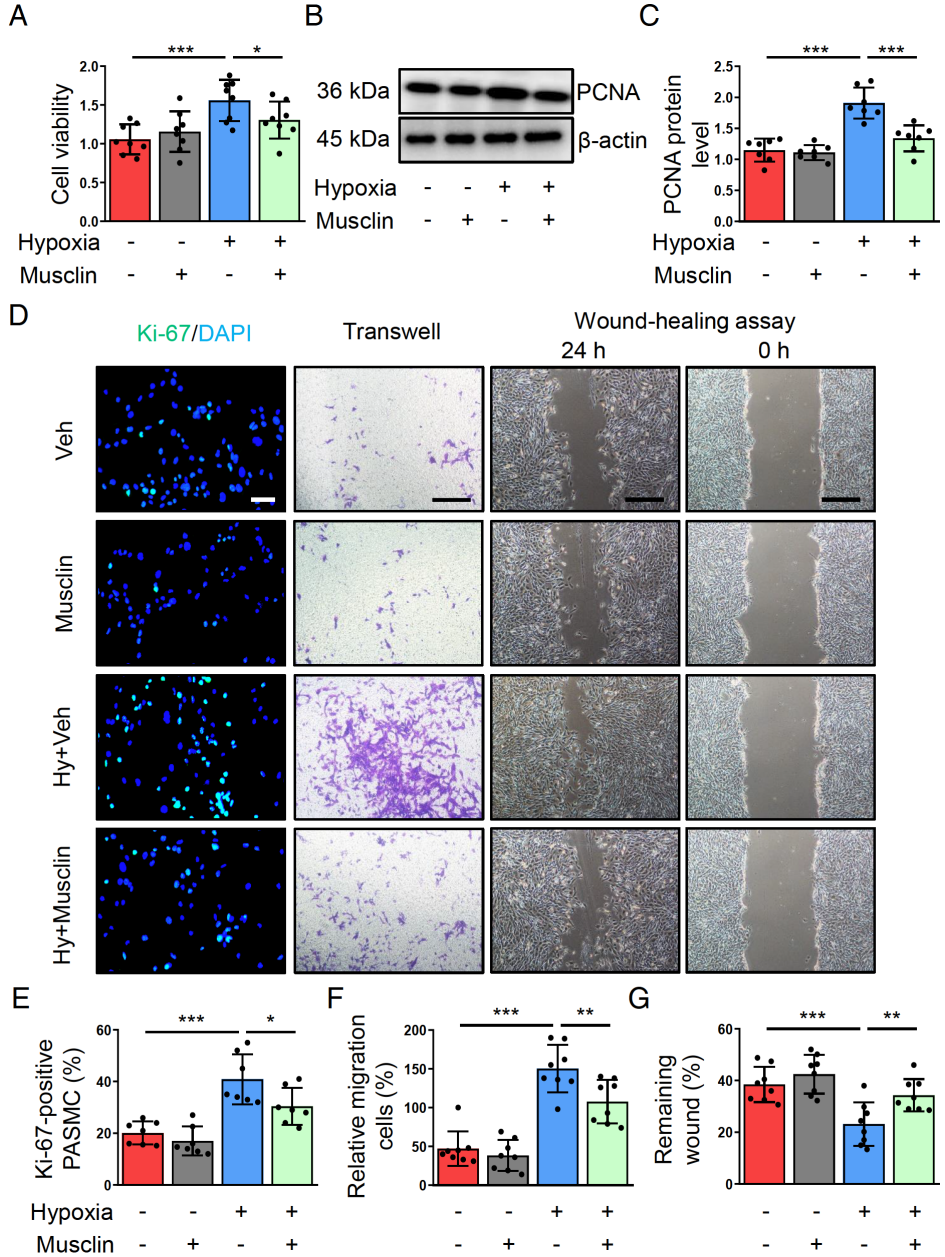
Figure 2 Musclin represses hypoxia-induced proliferation and migration of PASMCs (A) PASMCs were treated with vehicle or musclin (50 nM) and simultaneously incubated with or without hypoxia for 24 h. The viability of PASMCs from different groups was assessed via CCK-8 assay (n = 8). (B,C) Representative western blots and corresponding quantification of PCNA in PASMCs from different groups (n = 7). (D) PASMCs from different groups were stained with Ki-67 (green) and DAPI (blue). Representative images of Ki-67 staining (scale bar: 50 μm). The migration of PASMCs from different groups was measured via transwell and wound healing assays (scale bar: 200 μm). (E) The percentages of Ki-67-positive PASMCs in the different groups (n = 6). (F) Quantification of migrating cells from different groups (n = 8). (G) Quantification of wound healing rates in PASMCs from different groups (n = 8). *P < 0.05, **P < 0.01, and ***P < 0.001. Post hoc test for LSD in (A, C, E, F, and G).

Abnormal proliferation and migration of PASMCs constitute major events during PH development. Therefore, we also explored the effect of musclin on PASMCs. We first determined the optimal dose of musclin for treatment. The results showed that musclin inhibited the hypoxia-induced increase in PASMC viability in a dose-dependent manner ( Supplementary Figure S3). However, there was no significant difference between 50 nM and 100 nM. Therefore, we selected a dose of 50 nM for musclin treatment in subsequent experiments. In the present study, hypoxia markedly increased PASMC viability and the expression of proliferating cell nuclear antigen (PCNA), a marker of cell proliferation. Interestingly, musclin treatment significantly reduced the viability and PCNA expression of PASMCs ( Figure 2A–C). Next, we used Ki-67 immunofluorescence staining to analyze the proliferation of PASMCs. As expected, the hypoxia exposure-induced increase in the percentage of Ki-67-positive PASMCs was obviously attenuated after musclin treatment ( Figure 2D,E). Furthermore, we determined the migration of PASMCs via wound healing and transwell assays. The percentage of transmembrane PASMCs increased after hypoxia exposure, which was reversed by musclin treatment ( Figure 2D,F). Moreover, wound healing assay revealed that musclin could suppress hypoxia-induced cellular scratch closure ( Figure 2D,G). These results indicate that musclin represses hypoxia-induced proliferation and migration in PASMCs.

### Musclin inhibits hypoxia-induced glycolysis and oxidative stress in PASMCs

**Figure 3:**
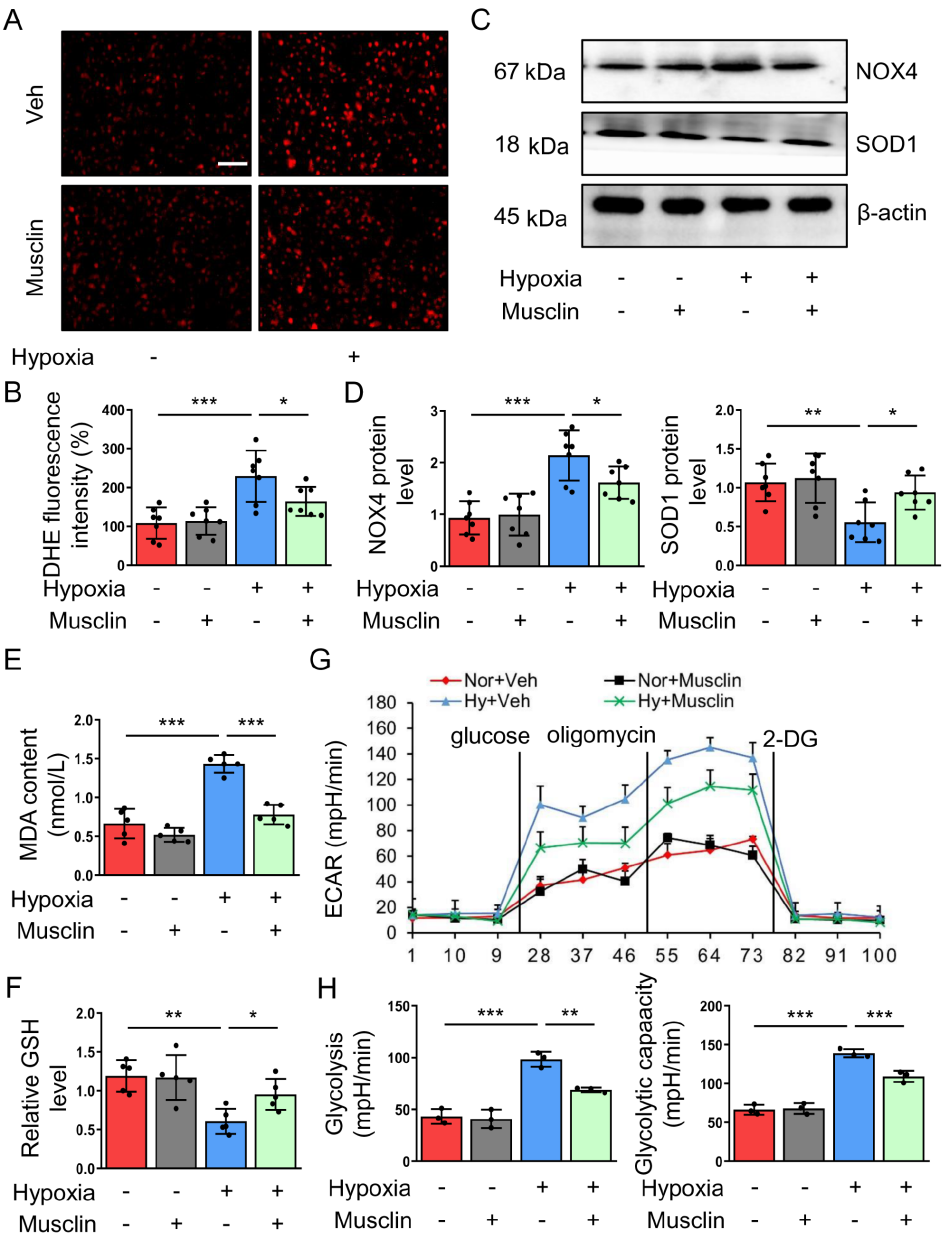
Figure 3 Musclin suppresses glycolysis and oxidative stress in hypoxia-challenged PASMCs (A,B) PASMCs from different groups were stained with DHE (red). Representative images and the corresponding intensity of DHE staining (n = 7). Scale bar: 50 μm. (C,D) Representative western blots and corresponding quantification of NOX4 and SOD1 in PASMCs from different groups (n = 7). (E,F) The MDA content and relative GSH level in the supernatant of PASMCs from different groups (n = 5). (G,H) Glycolysis and glycolytic capacity in PASMCs from different groups were determined by the ECAR (n = 3). *P < 0.05, **P < 0.01, and ***P < 0.001. Post hoc test for LSD in (B, D, E, F, and H).

Exercise plays a critical role in repressing oxidative stress [15], which has been confirmed to aggravate the proliferation and migration of PASMCs [16]. Thus, we investigated whether musclin also inhibits oxidative stress. We utilized DHE staining to determine the reactive oxygen species (ROS) level. The results revealed that the relative DHE fluorescence intensity was markedly increased in hypoxia-challenged PASMCs. However, the increase in DHE intensity was attenuated after musclin treatment ( Figure 3A,B). The downregulated antioxidant SOD1 and upregulated NOX4 are involved in the abnormal proliferation of vascular smooth muscle cells in atherosclerotic lesions [28]. Consistently, hypoxia caused decreased SOD1 expression but increased NOX4 expression in PASMCs. However, musclin significantly reversed the hypoxia-induced alterations in SOD1 and NOX4 expressions in PASMCs ( Figure 3C,D). MDA is a marker of lipid peroxidation and plays a critical role in oxidative stress and pulmonary hypertension [29]. GSH serves as an important intracellular antioxidant [30]. Hypoxia caused increased MDA level but decreased GSH level in PASMCs. However, these effects were markedly attenuated by treatment with musclin ( Figure 3E,F).

Glycolysis serves as a major source of ROS in vascular diseases [17]. Musclin is known to repress glucose utilization in adipose tissue and skeletal muscle [ 19, 20] . To explore whether musclin also modulates glucose metabolism in hypoxia-challenged PASMCs, we utilized ECAR experiments to analyze glycolytic capacity. In the present study, hypoxia exposure significantly increased glycolysis and glycolytic capacity in PASMCs, which was partly attenuated by musclin treatment ( Figure 3G,H). Thus, musclin may suppress hypoxia-induced proliferation and migration in PASMCs partly by inhibiting glycolysis and subsequent oxidative stress.

### Musclin inhibits glycolysis, oxidative stress, proliferation, and migration in hypoxia-challenged PASMCs in an NPR3-dependent manner

**Figure 4:**
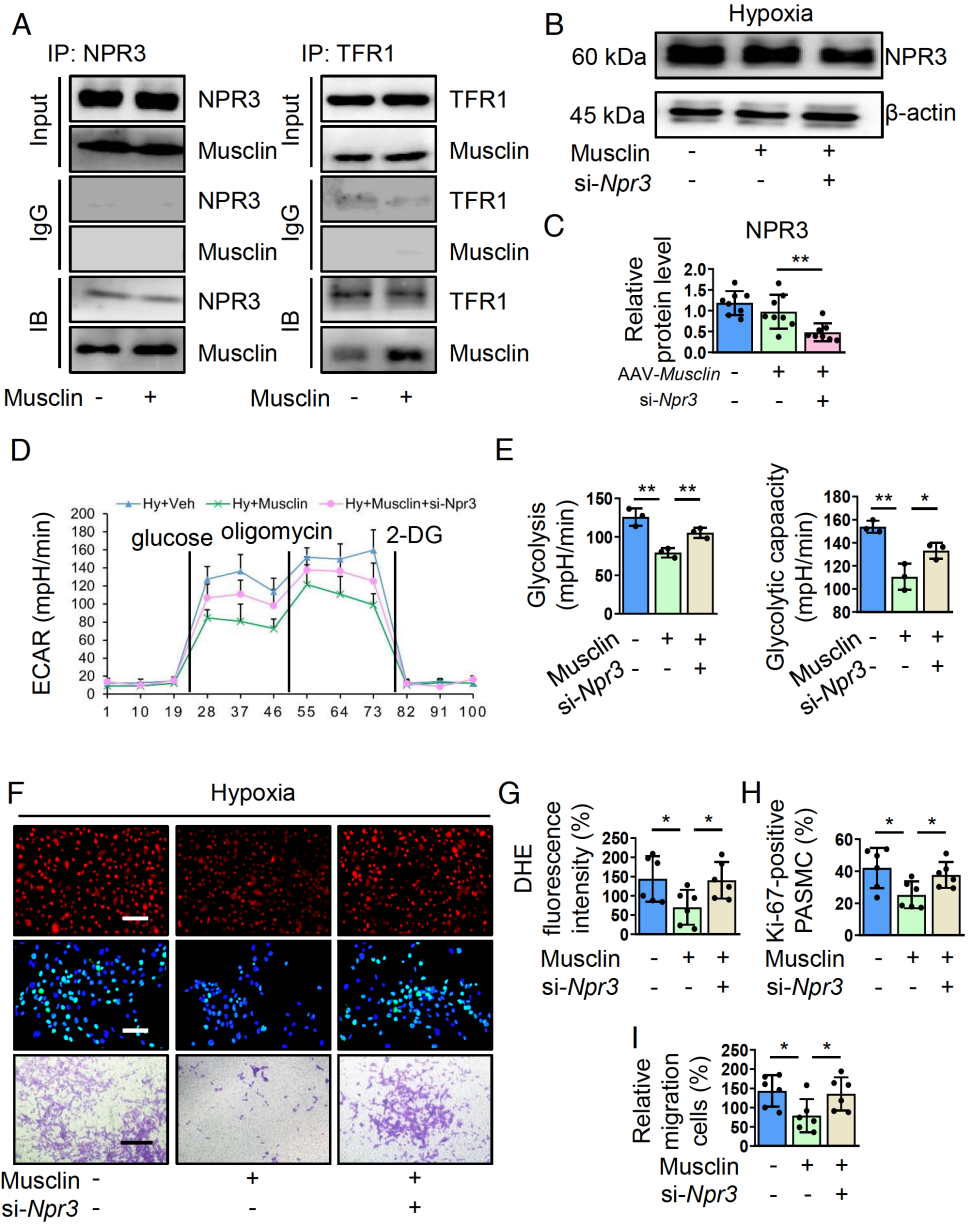
Figure 4 Musclin inhibits the proliferation and migration of hypoxia-stimulated PASMCs by binding to NPR3 (A) PASMCs were treated with musclin (50 nM) for 24 h and then subjected to co-IP analysis. (B,C) Representative western blots and corresponding quantification of NPR3 in PASMCs from different groups (n = 8). (D,E) Glycolysis and glycolytic capacity in PASMCs from different groups were determined by the ECAR (n = 3). (F) PASMCs from different groups were stained with DHE (red). Scale bar: 50 μm. PASMCs from different groups were stained with Ki-67 (green) and DAPI (blue). Scale bar: 50 μm. The migration of PASMCs from different groups was measured via transwell assay. Scale bar: 200 μm. (G) The relative intensity of DHE in PASMCs from different groups (n = 6). (H) The percentages of Ki-67-positive PASMCs in the different groups (n = 6). (I) Quantification of migrating cells from different groups (n = 6). *P < 0.05 and **P < 0.01. Post hoc test for LSD in (C, E, G, H, and I).

We next investigated the mechanism by which musclin regulates PASMCs. NPR3 and TFR1 are two well-known receptors within the cell membrane for musclin [ 10, 19] . We first utilized co-IP assay to assess the binding of musclin to TFR1 and NPR3 in PASMCs. The results showed that musclin could bind to both NPR3 and TFR1 in PASMCs. In addition, the interaction of musclin with both TFR1 and NPR3 was increased in musclin-treated PASMCs ( Figure 4A). The knockdown of *NPR3* by si- *Npr3* was confirmed in PASMCs ( Figure 4B,C). Subsequently, an ECAR assay and DHE immunofluorescence staining revealed that silencing of *NPR3* abolished the inhibitory effects of musclin on glycolysis and ROS generation in hypoxia-challenged PASMCs ( Figure 4D–G). A previous study confirmed that NPR3 inhibits human vascular muscle cell proliferation [31]. This finding prompted us to investigate whether NPR3 is also essential for musclin-mediated regulation of PASMCs. Ki-67 immunofluorescence staining and transwell assays revealed that attenuated proliferation and migration in hypoxia-challenged PASMCs induced by musclin were also enhanced after silencing of *NPR3* ( Figure 4F,H,I). However, *TFR1* silencing did not affect musclin-mediated regulation of glycolysis ( Supplementary Figure S4A) or proliferation ( Supplementary Figure S4B) in PASMCs. These data suggest that musclin regulates PASMCs through interaction with NPR3.

### mTORC1 inactivation is essential for musclin-mediated inhibition of proliferation and migration in PASMCs

**Figure 5:**
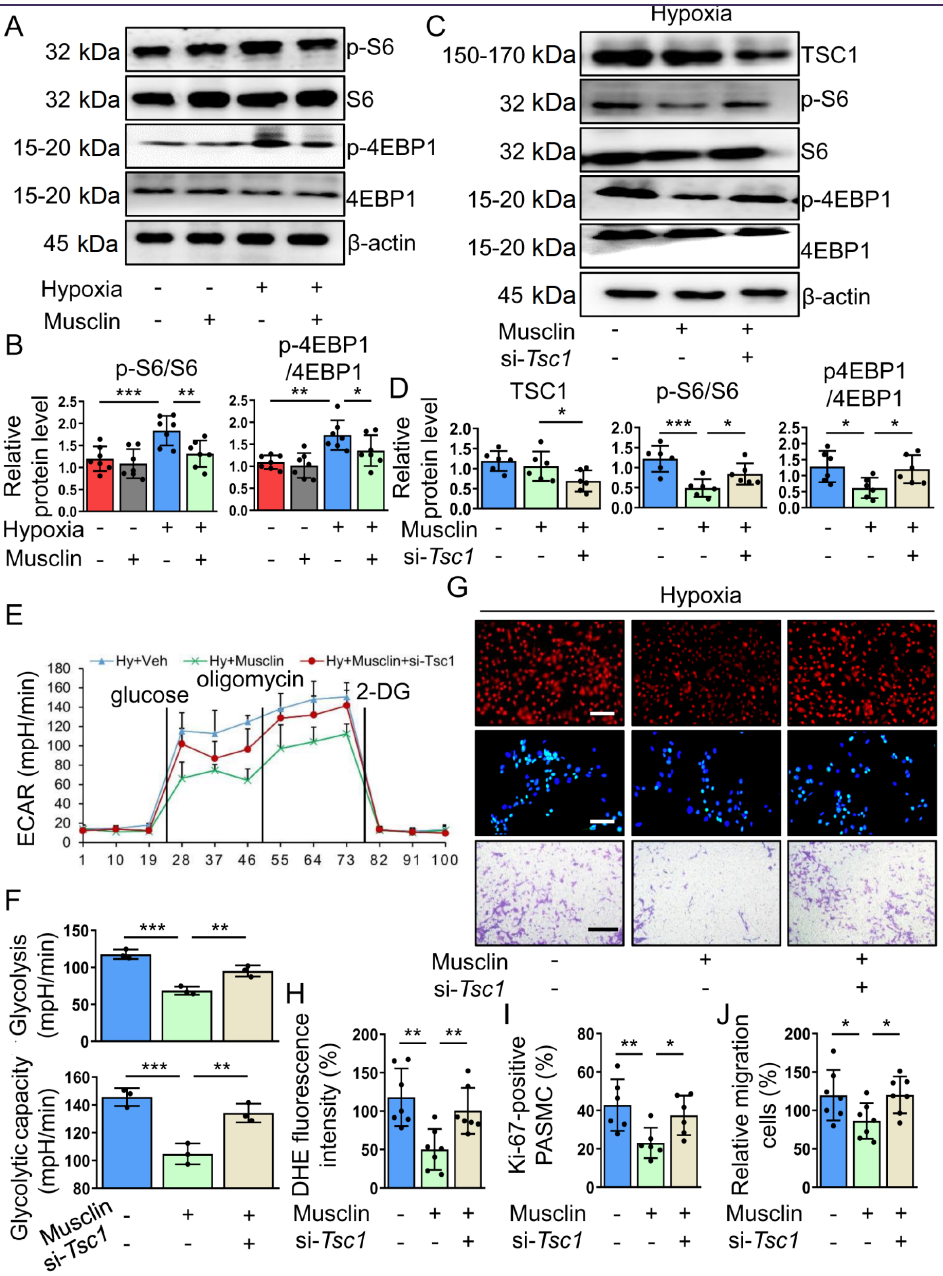
Figure 5 Musclin suppresses the proliferation and migration of hypoxia-stimulated PASMCs by inhibiting mTORC1 activity (A,B) PASMCs were treated with vehicle or musclin (50 nM) and simultaneously incubated with or without hypoxia for 24 h. Representative western blots and corresponding quantification of p-S6/S6 and p-4EBP1/4EBP1 in PASMCs from different groups (n = 7). (C,D) Representative western blots and corresponding quantification of TSC1, p-S6/S6, and p-4EBP1/4EBP1 in PASMCs from different groups (n = 6). (E,F) Glycolysis and glycolytic capacity in PASMCs from different groups were determined by the ECAR (n = 3). (G) PASMCs from different groups were stained with DHE (red). Scale bar: 50 μm. PASMCs from different groups were stained with Ki-67 (green) and DAPI (blue). Scale bar: 50 μm. The migration of PASMCs from different groups was measured via transwell assay. Scale bar: 200 μm. (H) The relative intensity of DHE in PASMCs from different groups (n = 7). (I) The percentages of Ki-67-positive PASMCs in the different groups (n = 6). (J) Quantification of migrating cells from different groups (n = 7). *P < 0.05, **P < 0.01, and ***P < 0.001. Post hoc test for LSD in (B, D, F, H, I, and J).

Activated by a series of stimuli, including hypoxia, stress, growth factors, and inflammatory factors, mTORC1 is known to promote the progression of PH [32]. Since mTORC1 activation is also closely related to glycolysis and oxidative stress in blood vessels [ 27, 33] , we next investigated the effect of musclin/NPR3 on mTORC1 signaling in PASMCs. Consistent with the findings of previous studies, mTORC1 activity was obviously increased in PASMCs after hypoxia, as reflected by the increased phosphorylation of S6 and 4EBP1, the two major effectors of mTORC1. However, the increase in mTORC1 activity in hypoxia-challenged PASMCs was retarded by musclin ( Figure 5A,B). TSC1 acts as an inhibitor of mTORC1 activity [34]. Therefore, we used si- *Tsc1* to reactivate mTORC1 in PASMCs after musclin treatment and observed elevated mTORC1 activity in musclin-treated PASMCs after silencing of *TSC1* ( Figure 5C,D). These results suggest that *TSC1* silencing can reverse the attenuated mTORC1 activity in PASMCs induced by musclin.

Next, we investigated whether recovering mTORC1 activity could abolish musclin-mediated inhibition of oxidative stress, glycolysis, proliferation, and migration in hypoxia-challenged PASMCs. ECAR and DHE staining revealed that glycolysis and ROS generation were both markedly increased in musclin-treated PASMCs after silencing of *TSC1* ( Figure 5E–H). Additionally, the suppressive effects of musclin on proliferation ( Figure 5G,I) and migration ( Figure 5G,J) in hypoxia-challenged PASMCs were abolished after reactivating mTORC1 via si- *Tsc1* transfection.

Next, we determined the role of NRP3 or TFR1 in musclin-mediated regulation of mTORC1. An siRNA strategy revealed that the attenuated phosphorylation levels of S6 and 4EBP1 in hypoxia-challenged PASMCs induced by musclin were significantly elevated after silencing of *NPR3* ( Supplementary Figure S5A,B). AKT serves as a canonical upstream kinase of mTORC1 and can be activated by hypoxia to induce mTORC1 activation in PASMCs [35]. Our current study demonstrated that AKT phosphorylation was also inhibited by musclin in hypoxia-stimulated PASMCs. Interestingly, the attenuated AKT phosphorylation induced by musclin was also reversed by si- *Npr3* ( Supplementary Figure S5A,B). However, silencing of *TFR1* did not affect musclin-mediated regulation of mTORC1 activity in PASMCs ( Supplementary Figure S4C). Overall, we conclude that musclin represses the proliferation and migration of PASMCs by binding to NPR3 and subsequently reducing AKT/mTORC1 activity.

### Musclin ameliorates MCT-induced PH by reducing mTORC1 activity in PAs

**Figure 6:**
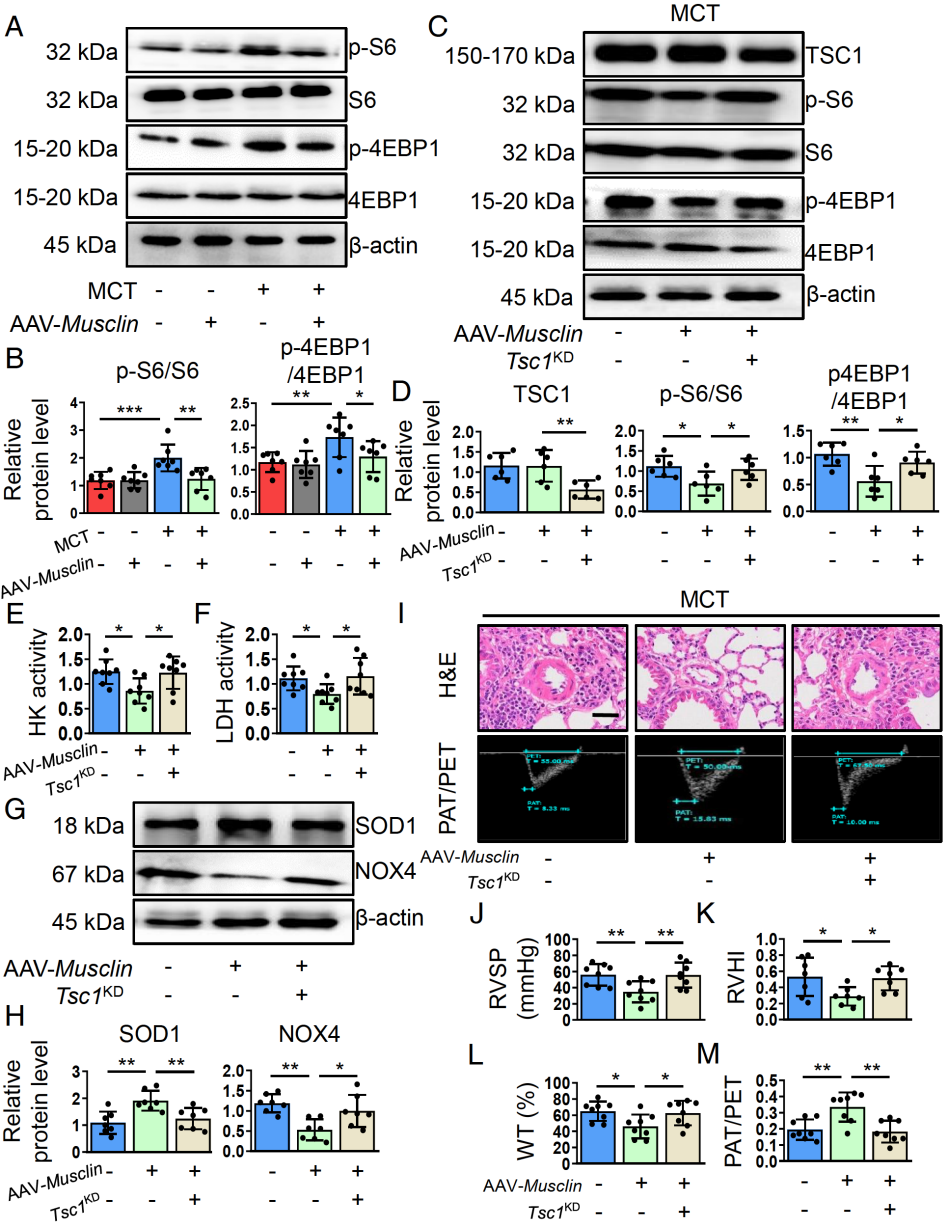
Figure 6 Skeletal muscle-derived musclin ameliorates MCT-induced PH by reducing mTORC1 activity in Pas (A,B) Representative western blots and corresponding quantification of p-S6/S6 and p-4EBP1/4EBP1 in PAs from mice in different groups (n = 7). (C,D) Representative western blots and corresponding quantification of TSC1, p-S6/S6, and p-4EBP1/4EBP1 in PAs from mice in different groups (n = 6). The activities of (E) HK and (F) LDH in the PAs of the mice in the different groups (n = 8). (G,H) Representative western blots and corresponding quantification of SOD1 and NOX4 in PAs from mice in different groups (n = 6). (I) Representative H&E staining images (scale bar: 20 μm) and echocardiography images of the mice in the different groups. Quantification of (J) RVSP (n = 8), (K) RVHI (n = 7), (L) the percentage of pulmonary wall thickness/total thickness (n = 8), and (M) PAT/PET (n = 8) in the mice in the different groups. *P < 0.05, **P < 0.01, and ***P < 0.001. Post hoc test for LSD in (B, D, E, F, H, J, L, and M). Post hoc test for Dunnett’s T3 test in (K).

Since mTORC1 is involved in musclin-mediated regulation of PASMCs, we next explored whether mTORC1 is also involved in musclin-mediated protection against PH *in vivo*. We first dissected the PAs and determined the change in mTORC1 activity following AAV- *Musclin* transfection in skeletal muscle. We found that pulmonary vascular mTORC1 activity, reflected by the phosphorylation of S6 and 4EBP1, was significantly increased in MCT-treated mice, whereas this change was reversed following musclin overexpression ( Figure 6A,B). To investigate the role of mTORC1 inactivation in musclin-mediated modulation of PH, we utilized *Tsc1* ^KD^ mice to recover mTORC1 activity in PAs induced by musclin ( Figure 6C,D), as described in our previous study [36]. Oxidative stress and excessive glycolysis are important regulators of PH development [ 16, 18] . Our results revealed decreased HK and LDH activity, increased SOD1 expression, and decreased NOX4 expression in MCT-treated PAs of musclin-overexpressing mice. However, these changes were reversed in *Tsc1* ^KD^ mice ( Figure 6E–H). Furthermore, we analyzed the role of musclin in PH progression in *Tsc1* ^KD^ mice. Our results revealed that mTORC1 reactivation by *TSC1* silencing abolished musclin-mediated suppression of RVSP, RVHI, and hyperplasia of peripheral PAs in MCT-treated mice ( Figure 6I–L). Moreover, the increased PAT/PET ratio following musclin overexpression in skeletal muscle was also reduced in *Tsc1* ^KD^ mice ( Figure 6I,M). Consistent with these findings, the inhibition of fibrosis around the PA and vascular muscularization by musclin was also impaired in *Tsc1* ^KD^ mice ( Supplementary Figure S6). These data indicate that musclin inhibits MCT-induced PH progression in an NPR3/AKT/mTORC1-dependent manner.

## Discussion

The current study revealed that both the production of musclin in skeletal muscle and the secretion of musclin from skeletal muscle into the circulation were downregulated in a mouse PH model. External expression of musclin in skeletal muscle by AAV-6 transfection ameliorated pulmonary vascular wall thickening and right ventricular hypertrophy. *In vitro*, musclin inhibited hypoxia-induced glycolysis, oxidative stress, proliferation, and migration in PASMCs. Mechanistically, musclin suppressed the phosphorylation of AKT and downstream mTORC1 activation through binding to NPR3. These findings reveal a novel paracrine role of skeletal muscle in preventing PH development through the modulation of the production and secretion of musclin.

Several studies have indicated that exercise can improve the symptoms and clinical outcomes of PH patients [ 4, 5] . Specifically, several lines of evidence suggest that skeletal muscle-secreted myokines after exercise mediate these protective effects [ 6, 7] . For example, a lower level of the myokine irisin in PH patients is associated with higher mean pulmonary artery pressure, mortality, and adverse hemodynamic status [6]. Another myokine, follistatin-like 1, has been reported to inhibit the proliferation and migration of PASMCs in a mouse PH model [7]. Originally found to be located in young osteocytes and osteoblasts, musclin is known to regulate osteoblast function and bone development and formation [37]. Additionally, accumulating evidence indicates that musclin also serves as a secretory factor released by skeletal muscle to perform various functions, such as modulating metabolism [19]. The role of musclin in vascular diseases has recently attracted increasing attention. For example, vascular inflammation, a core event in atherosclerosis-associated diseases, is markedly suppressed by musclin [13]. Patients with hypertension and transcatheter aortic valve implantation also display decreased musclin level in peripheral blood [ 11, 12] . These studies suggest a potential protective role of musclin in vascular diseases. Consistent with these studies, our current study revealed that the level of musclin was decreased in both skeletal muscle and circulation in a mouse PH model. Overexpression of musclin in skeletal muscle ameliorated vascular remodeling of PAs and right ventricular hypertrophy. These data suggest that musclin may act as a paracrine myokine derived from skeletal muscle to exert a protective role against PH.

Excessive proliferation and migration of PASMCs are crucial events during PA remodeling and PH progression [3]. Thus, we determined whether the vascular protective effects of musclin are achieved via the regulation of PASMCs. In the present study, we observed that the hypoxia-induced proliferation and migration of PASMCs were obviously repressed by musclin *in vitro*. This finding is also consistent with the anti-proliferative roles of musclin in other cell types [14]. In response to hypoxic conditions, metabolic reprogramming-induced oxidative stress causes PASMC proliferation and aggravates PH development [16]. Enhanced glucose metabolism is an important hallmark of metabolic reprogramming. Consistently, we also observed accelerated oxidative stress and increased glycolysis in the hypoxia-challenged PASMCs and PAs of MCT-treated mice. Musclin significantly suppressed oxidative stress and glycolysis in PASMCs after hypoxic stimulus. More importantly, the external expression of musclin in skeletal muscle could also repress oxidative stress and glucose metabolism in PAs from an MCT-induced mouse model of PH. The role of musclin in impairing glycolysis in PASMCs is also consistent with musclin-mediated repression of glucose uptake and metabolism reported in previous studies [ 19, 20] . These findings suggest that musclin may inhibit hypoxia-induced proliferation, the migration of PASMCs and MCT-induced PH development partly through mitigating glycolysis and oxidative stress.

As a secretory protein that functions between different tissues, musclin usually exerts its biological effects by binding to corresponding membrane receptors. NPR3 and TFR1 are two well-known receptors for musclin [ 10, 19] . However, the binding of musclin to NPR3 or TFR1 in PASMCs remains unclear. In terms of the molecular mechanism underlying the regulation of PASMCs by musclin, the interaction of musclin with NPR3 or TFR1 was assessed via co-IP. Interestingly, the interactions of musclin-NPR3 and musclin-TFR1 were both enhanced in PASMCs after musclin treatment. A previous study demonstrated that NPR3 is involved in inhibiting the proliferation of human vascular smooth muscle cells [31]. Consistently, our further study used siRNA strategy to downregulate NPR3 or TFR1 expression and revealed that NPR3, rather than TFR1, is responsible for the musclin-mediated inhibition of glycolysis, oxidative stress, proliferation, and migration in PASMCs. These findings highlight the crucial role of NPR3 in repressing glycolysis, oxidative stress, proliferation, and migration via musclin in hypoxia-challenged PASMCs.

Hypoxia activates a variety of stress-associated molecules, including protein kinases and transcription factors. Among these stress sensors, mTORC1 plays crucial roles in hypoxia-induced proliferation, migration of PASMCs, and PH development [35]. In the present study, we found that hypoxia-induced mTORC1 activation in PASMCs was markedly attenuated after musclin treatment. Suppression of mTORC1 activation was also observed in PAs of the MCT-induced PH model after overexpression of musclin in skeletal muscle. These results suggest that skeletal muscle-derived musclin may inhibit mTORC1 activity in the PASMCs of MCT-treated animals via a paracrine mechanism. By using a *TSC1*-knockdown mouse model or a si- *Tsc1* strategy to reactivate mTORC1 [34], the inhibitory effects of musclin on the proliferation and migration of hypoxia-challenged PASMCs and MCT-induced PH development were both impaired. In addition, increased mTORC1 activity is related to increased glycolysis in PASMCs [38]. In this study, restoring mTORC1 activity by knocking down *TSC1* abolished the inhibitory effects of musclin on glucose utilization and oxidative stress both *in vivo* and *in vitro*. Since AKT serves as an important upstream kinase that positively regulates mTORC1 activity in hypoxia-challenged PASMCs [35], we also confirmed that musclin could inhibit the phosphorylation of AKT. However, the modulatory effect of musclin on mTORC1 activity in PASMCs was reversed after the silencing of *NPR3* but not *TFR1*. Therefore, musclin protects against PH through binding to NPR3 and subsequently attenuating AKT/mTORC1 activity in PASMCs.

**Figure 7:**
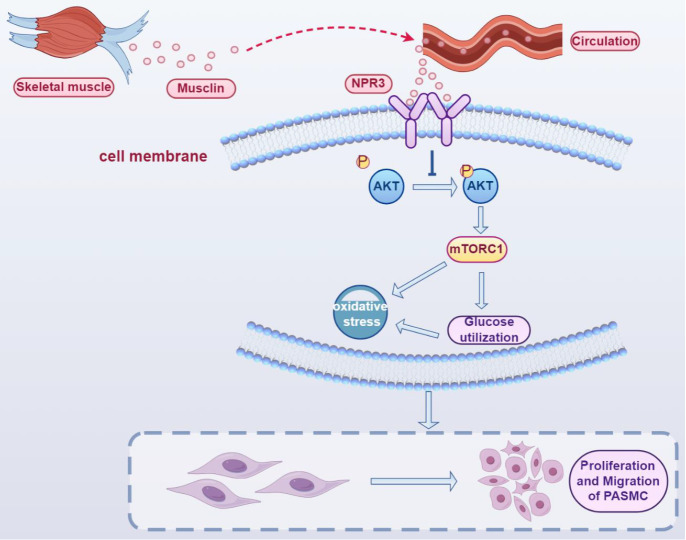
Figure 7 Skeletal muscle-derived musclin suppresses the proliferation and migration of PASMCs and pulmonary hypertension partly by attenuating glycolysis and oxidative stress in an NPR3/AKT/mTORC1-dependent manner Overexpression of musclin in skeletal muscle via AAV6 transfection results in increased musclin release into the circulation from skeletal muscle. Musclin suppresses AKT phosphorylation and thus inhibits subsequent mTORC1 activation by binding to NPR3. Decreased AKT/mTORC1 activity subsequently attenuates glycolysis, oxidative stress, proliferation, and migration in PASMCs, thereby ameliorating PH progression.

In conclusion, we revealed that the levels of musclin in both skeletal muscle and peripheral blood were decreased in the MCT-induced mouse PH model. AAV-6-mediated external overexpression of musclin in skeletal muscle was released into the circulation, inhibited AKT/mTORC1 activity and subsequent glycolysis, oxidative stress, proliferation, and migration in PASMCs by binding to NPR3, thereby protecting against PH development ( Figure 7).

## Supporting information

24654Supplementary_Figures

## Supplementary Data

Supplementary data is available at *Acta Biochimica et Biophysica Sinica* online.
