# Supplementary material for: Skeletal muscle-derived musclin attenuates glycolysis, oxidative stress, and pulmonary hypertension through the NPR3/AKT/mTORC1 pathway: Musclin in pulmonary hypertension
Source: Acta Biochim Biophys Sin (Shanghai). 2024 Dec 4;57(6):981–94. doi: 10.3724/abbs.2024214 (PMC12247140; doi:10.3724/abbs.2024214)
Supplement: 24654Supplementary_Figures [file 24654Supplementary_Figures.pdf]

Figure S1

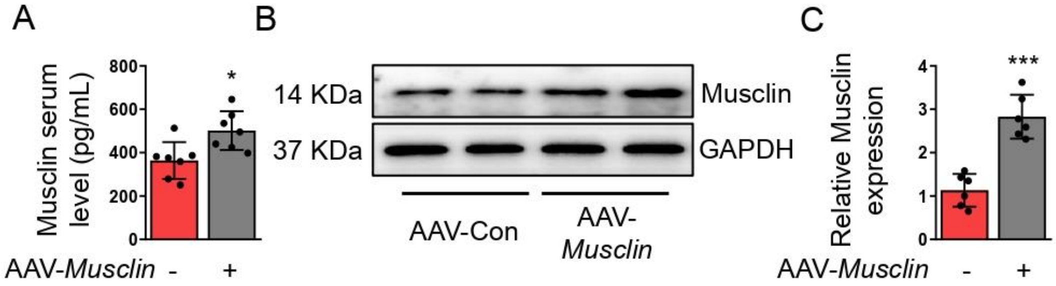

Supplementary Figure S1. AAV-*Musclin* induces increased musclin level in both serum and skeletal muscle (A) The serum musclin level in mice of different groups was determined by ELISA (n=7). (B,C) Representative western blots and corresponding quantification of musclin expression in right quadricep muscle from mice of different groups (*n*=6). *P<0.05 and ***P<0.001.

Figure S2

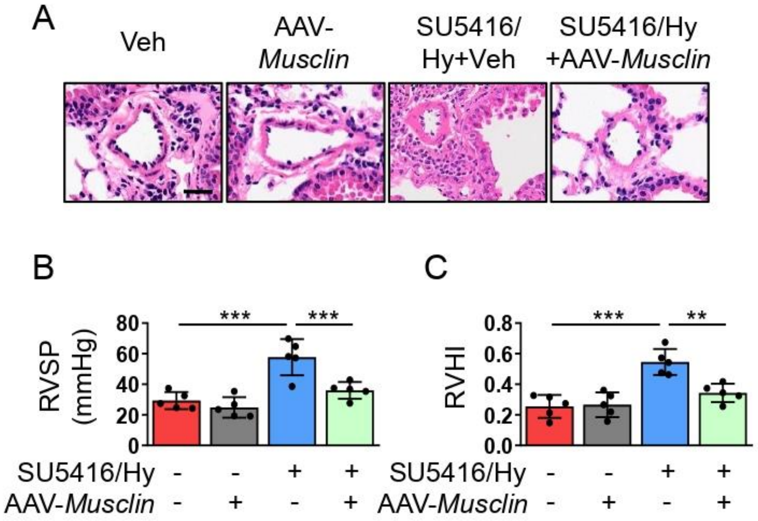

Supplementary Figure S2. Musclin reverses pulmonary arterial remodeling and right ventricular hypertrophy in SU5416/Hypoxia-induced mouse PH model (A) Representative H&E staining images (Scale bar: 20 μm) in mice of different groups. Quantification of (B) RVSP (*n*=5) and (C) RVHI (*n*=5) in mice of different groups. ***P*<0.01 and ****P* <0.001.

Figure S3

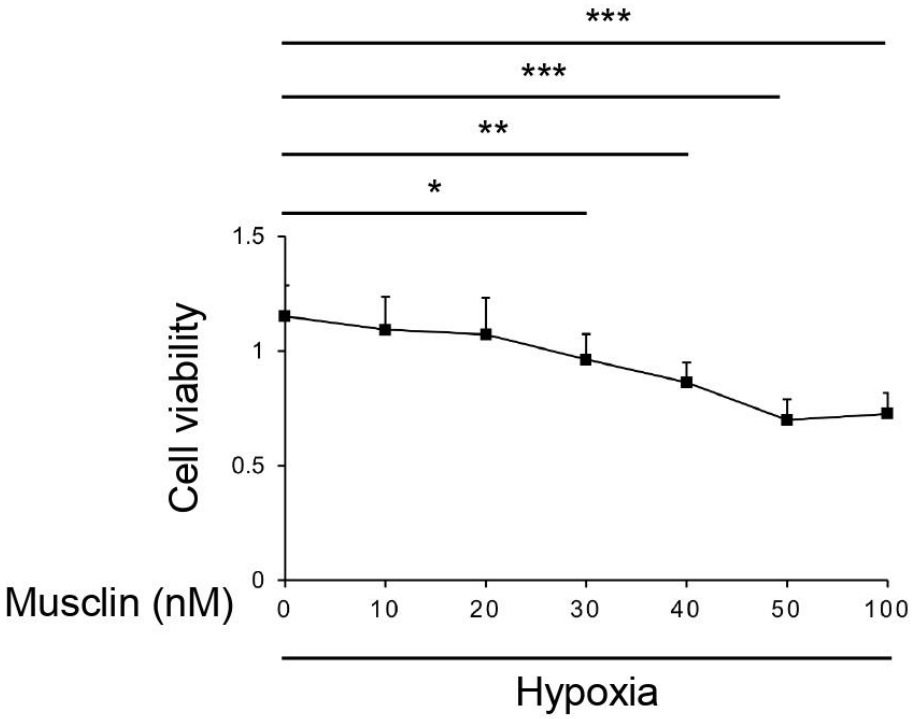

Supplementary Figure S3. Musclin inhibits hypoxia-induced increase in PASMC viability in a dose-dependent manner PASMCs were treated with musclin (0, 10, 20, 30, 40, 50, or 100 nM) and simultaneously incubated with hypoxia for 24 h. The viability of PASMCs from different groups was assessed by CCK-8 assay (*n*=5). *$P<0.05$, **$P<0.01$, and ***$P<0.001$.

Figure S4

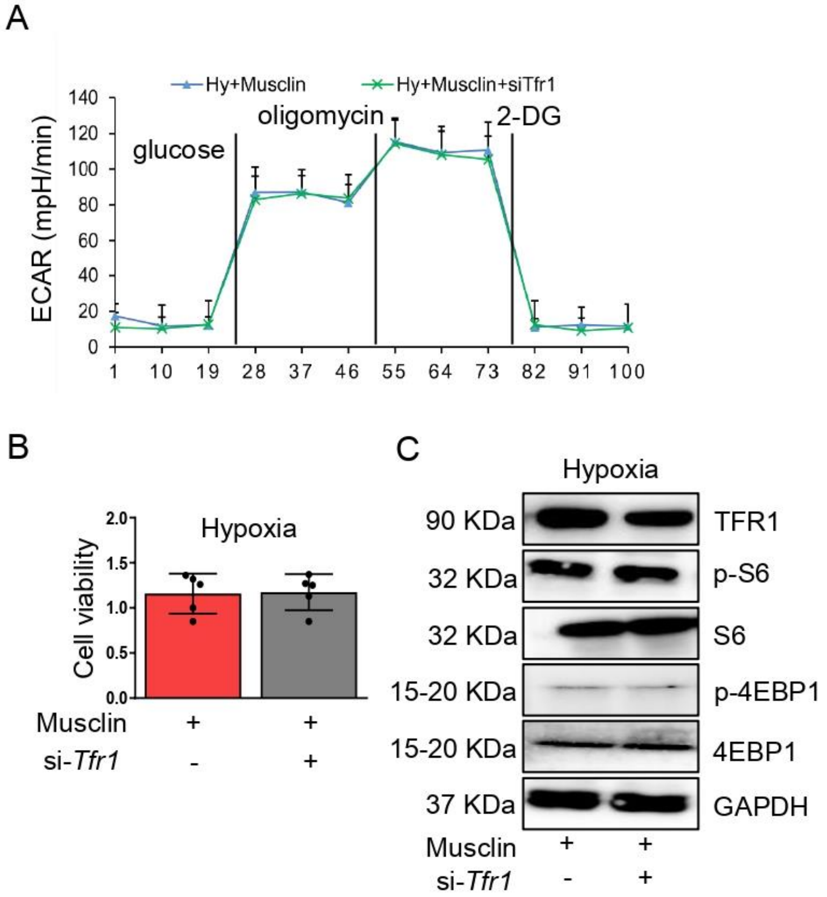

Supplementary Figure S4. *TFR1* silencing has no effect on musclin-mediated inhibition of glycolysis, proliferation, and mTORC1 activity in hypoxia-challenged PASMCs (A) Glycolysis and glycolytic capacities in PASMCs of different groups were determined by ECAR. (B) The relative viability of PASMCs in different groups was assessed by CCK-8 assay (*n*=5). (C) Representative western blots of TFR1, p-S6/S6 and p-4EBP1/4EBP1 in PASMCs from different groups.

Figure S5

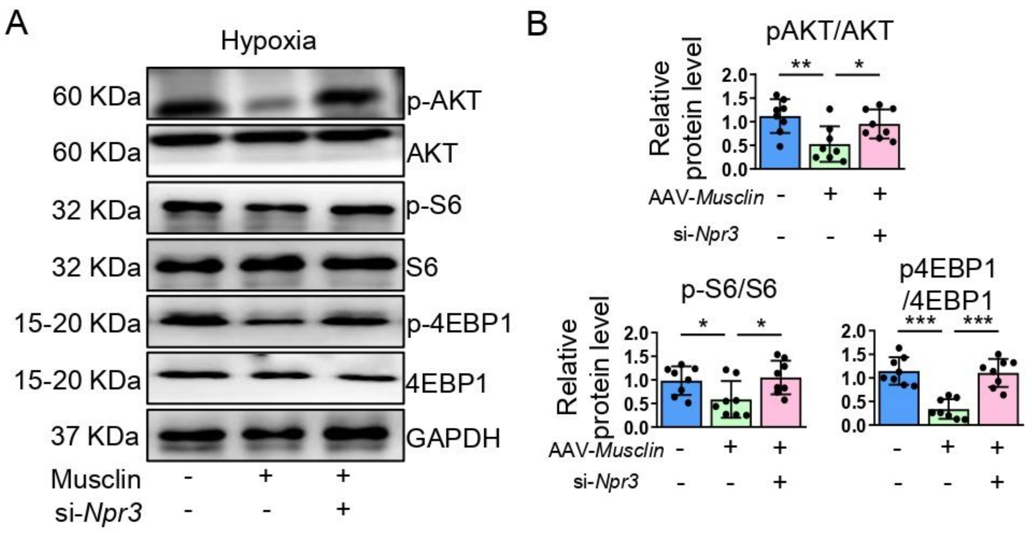

Supplementary Figure S5. *NPR3* silencing abolishes musclin-mediated inhibition of AKT/mTORC1 in hypoxia-challenged PASMCs (A,B) Representative western blots and corresponding quantification of p-AKT/AKT, p-S6/S6, and p-4EBP1/4EBP1 in PASMCs from different groups (*n*=8). **P* < 0.05, ***P* < 0.01, and ****P* < 0.001.

Figure S6

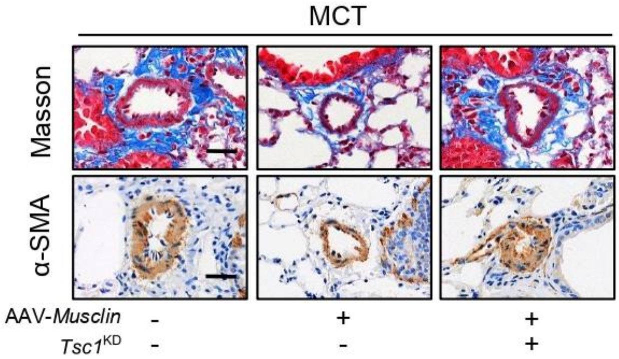

Supplementary Figure S6. Musclin represses fibrosis around PA and vascular muscularization by reducing mTORC1 activity Representative masson staining images (Scale bar: 20 μm) and immunohistochemical staining images of α-SMA (Scale bar: 20 μm) in mice of different groups.
